# Delta-6-desaturase gene polymorphism is associated with lipoprotein oxidation *in vitro*

**DOI:** 10.1186/1476-511X-12-80

**Published:** 2013-05-30

**Authors:** Tiina Solakivi, Tarja Kunnas, Olli Jaakkola, Jaana Renko, Terho Lehtimäki, Seppo T Nikkari

**Affiliations:** 1 Department of Medical Biochemistry, University of Tampere Medical School, Tampere, Finland; 2 Institute of Medical Technology, University of Tampere, Tampere, Finland; 3 Fimlab Laboratories, Tampere, Finland

## Abstract

**Background:**

Oxidative modification of low-density lipoprotein (LDL) is a key event in the oxidation hypothesis of atherogenesis. We have previously shown that HDL does not protect LDL from oxidation *in vitro*, but is in fact oxidized fastest of all lipoproteins due to its rich polyunsaturated fatty acid (PUFA) composition, which is oxidation promoting. Evidence has accumulated to show that in addition to diet, common polymorphisms in the fatty acid desaturase (FADS) gene cluster have very marked effects on human PUFA status. There is a deletion [T/-] in the promoter region of the Δ^6^ –desaturase gene (FADS2, rs 3834458), which has a direct inhibitory influence on production of PUFA from linoleic and alpha-linolenic acid. To investigate the possible role of rs 3834458 in lipoprotein modification, oxidation of LDL with HDL_2_ or HDL_3_ were analyzed from plasma of 58 free-living individuals.

**Results:**

Total eicosapentaenoic acid and arachidonic acid were significantly decreased in plasma from the 10 subjects homozygous for the deletion in FADS2 rs 3834458. When the isolated LDL and HDL_2_ were subjected to Cu^2+^-induced oxidation, these subjects showed decreased rate of appearance (p = 0.027) and the final concentration of conjugated dienes (p = 0.033) compared to the other genotypes. For oxidation of LDL with HDL_3_, the final concentration of conjugated dienes was also significantly decreased in subjects with [−/−] compared with [T/T] and [T/-] (p = 0.034).

**Conclusion:**

We conclude that FADS2 genotype may play a role in peroxidation susceptibility of lipoproteins.

## Background

Dietary and endogenously produced fatty acids are known to modulate the metabolism of lipids and lipoproteins and therefore also to be involved in cardiovascular and metabolic diseases [1]. Polyunsaturated fatty acids (PUFA) are classified into two families, the n–6 and n–3 series.

**Figure 1:**
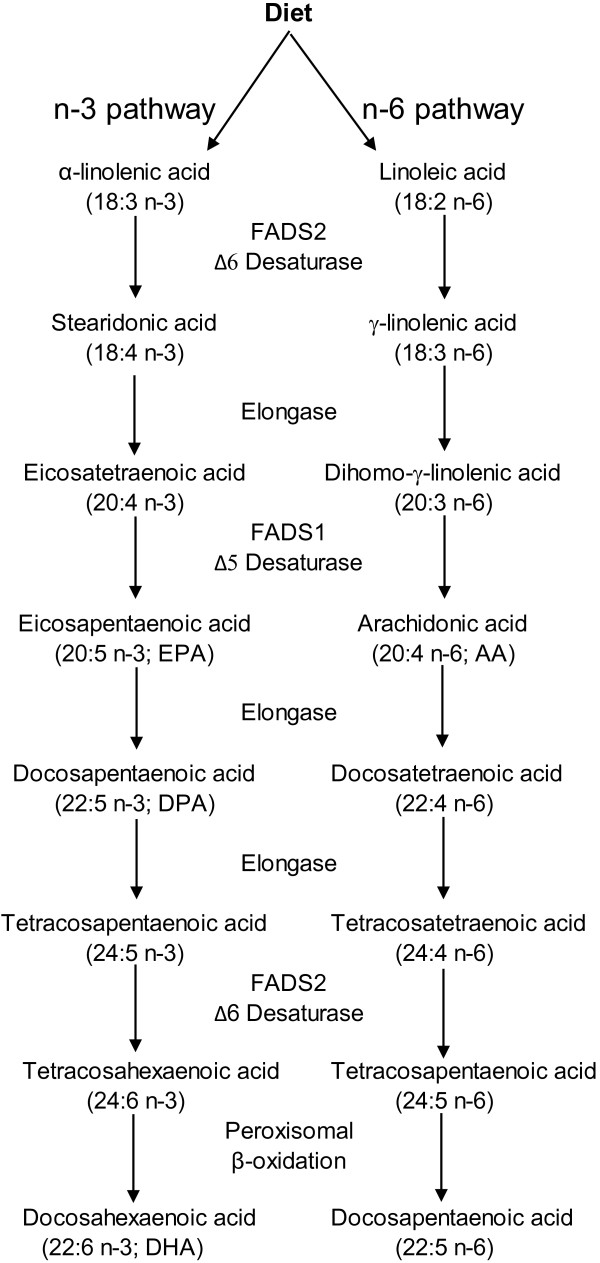
**Metabolic conversions of dietary linoleic and α-linolenic acids by Δ6 and Δ5 desaturases and elongases to their respective metabolites (modified from **[7]**).**

Linoleic acid (18:2 n-6) and α-linolenic acid (18:3 n-3) have become known as essential fatty acids (EFA) because they cannot be synthesized de novo by mammals, including humans, but are nevertheless necessary for proper physiological functioning [1]. The human body can then modify fatty acids by Δ6 and Δ5 desaturases and elongases to their respective metabolites along the pathways shown in Figure 1 to meet the metabolic needs. There is a common deletion [T/-] in the promoter region of the Δ6 desaturase gene (FADS2, rs 3834458) that has been shown to lead to decreased plasma levels of arachidonic acid and eicosapentaenoic acid [2,3]. Polyunsaturated fatty acids (PUFA) may influence inflammation, as they are precursors to eicosanoids [4]. Especially the eicosanoids that are derived from arachidonic acid (20:4n–6), a metabolite of linoleic acid (18:2n–6), have mainly pro-inflammatory effects [4]. In addition to being precursors to eicosanoids, PUFA are readily auto-oxidized in the presence of oxygen. This process is accelerated by the presence of trace metals and resisted by chelating agents and antioxidants. Thus PUFA play roles both in regulation of inflammation, and as direct targets for peroxidation through their double bonds that promote oxidation. PUFA play an important role in atherosclerosis through regulation of cholesterol transport by lipoproteins, and oxidative modification of low-density lipoprotein (LDL) is a key event in the oxidation hypothesis of atherogenesis [5]. We have previously shown that HDL does not protect LDL from oxidation *in vitro*, but is in fact oxidized fastest of all lipoproteins due to its rich polyunsaturated fatty acid (PUFA) composition, which is oxidation promoting [6]. In the present study, we tested the effect of FADS2 rs 3834458 on lipoprotein oxidation in vitro, in plasma derived from healthy subjects.

## Results

Background characteristics of the subjects participating in the study are shown in Table 1. The overall frequencies for the FADS2 insertion (T)/deletion (−) genotype groups in the study population were 0.24 for T/T, 0.59 for T/-, and 0.17 for −/−. The genotype distribution followed Hardy–Weinberg equilibrium.

**Table 1:** Characteristics of the study subjects (n = 58)

Age (years)	39.3 ± 10.6
Body mass index (kg/m^2^)	24.4 ± 3.6
Total cholesterol (mmol/l)	5.47 ± 0.97
Triacylglycerol (mmol/l)	1.35 ± 0.71
HDL cholesterol (mmol/l)	1.65 ± 0.38
LDL cholesterol (mmol/l)	3.21 ± 0.96

**Figure 2:**
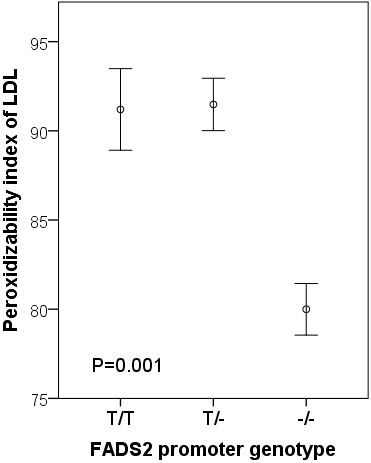
**Means (SEM) of peroxidizability index of LDL, according to FADS2 rs 3834458 gene variants.** −/−, homozygous for the variant (deletion) allele.

**Figure 3:**
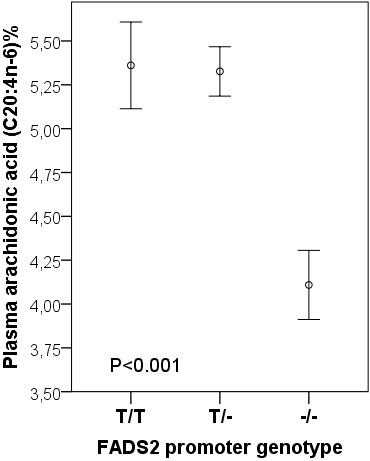
**Means (SEM) of plasma arachidonic acid proportions, according to FADS2 rs 3834458 gene variants.** −/−, homozygous for the variant (deletion) allele.

**Figure 4:**
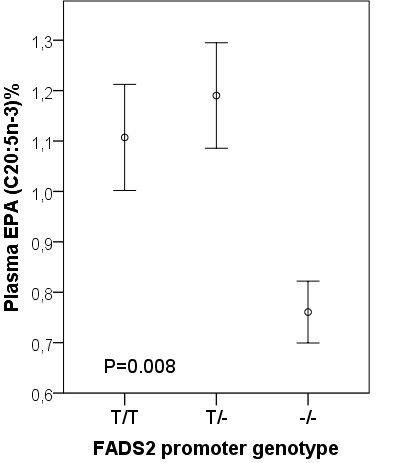
**Means (SEM) of plasma eicosapentaenoic acid (EPA) proportions according to FADS2 rs 3834458 gene variants.** −/−, homozygous for the variant (deletion) allele.

Compared with subjects with [T/T] or [T/-] in FADS2 rs 3834458, the peroxidizability index (Figure 2), the proportions of plasma total arachidonic acid (20:4 n-6) (Figure 3) and eicosapentaenoic acid (20:5 n-3) (Figure 4) were significantly decreased in the subjects homozygous for the deletion [−/−]. Also docosapentaenoic acid (22:5 n-3) was similarly decreased (p = 0.016) but not docosahexaenoic acid (22:6 n-3)(p = NS). The decreases for peroxidizability index, arachidonic acid, eicosapentaenoic acid and docosapentaenoic acid remained significant after adjusting for age and gender (p < 0.001, p < 0.001, p = 0.008 and p = 0.010 respectively). Plasma levels of linoleic acid and α-linolenic acid did not correlate with the FADS2 rs 3834458 polymorphism (p = NS).

Genotypes [T/T] and [T/-] were combined for statistical analyses of the oxidation study since they showed similar profiles for their effects on PUFA proportions. When mixtures of isolated LDL and HDL_2_ were subjected to Cu^2+^-induced oxidation, the subjects with [−/−] showed decreased rate of appearance (p = 0.027) and the final concentration (P = 0.033) of conjugated dienes, compared with [T/T] and [T/-] (Table 2). The findings remained significant after adjusting for age and gender. When isolated HDL_3_ was subjected to Cu^2+^-induced oxidation with LDL, a similar trend was observed in oxidation rate, but only the final concentration of conjugated dienes was significantly decreased in subjects with [−/−] compared with [T/T] and [T/-] (p = 0.034; p = 0.031 after adjusting for age and gender) (data not shown).

**Table 2:** **Kinetic parameters of LDL + HDL**_**2**_**oxidation in healthy subjects according to FADS2 genotype**

**Genotype**	**TT and T –**	**– –**	**P**	P*
**N**	**48**	**10**		
**LDL + HDL**_**2**_				
Lag time (min)	56.1 ± 6.2	57 ± 5.4	0.764	0.296
Rate (μmol/l/min)^a^	0.685 ± 0.075	0.656 ± 0.085	0.027	0.027
Max (nmol/mg)^b^	777 ± 50	735 ± 74	0.033	0.034

Values are mean ± SD. ^a^ Rate means maximal formation rate of conjugated dienes during oxidation. Calculation of the diene concentration is based on ϵ_234 nm_ = 29500 l/mol/cm of the conjugated dienes. ^b^ Max is the maximal amount of dienes produced per mg of LDL protein.

*; adjusted for age and gender.

## Discussion

The overall frequencies for the FADS2 rs 3834458 insertion (T)/deletion (−) genotype groups in the study population were 0.24 for T/T, 0.59 for T/-, and 0.17 for −/−. These differed from the KOALA study (the Netherlands) and the LISA study (Germany), where the frequencies were 0.46, 0.43, 0.11 and 0.47, 0.44, 0.09, respectively [8]. In both of these studies, the FADS2 gene rs3834458 was typed with MALDI-TOF-MS. On the other hand, in subjects from Costa Rica, by using a variation of the allele-specific assay, the corresponding frequencies were 0.29, 0.47 and 0.23 [3], which are more in line with our results. The difference in frequency in our study compared to the KOALA and LISA studies might be due to a different method of analysis, or to a difference in the study populations [8]. However, prior to analysis, we confirmed the products of our PCR primers to be specific for FADS2 rs 3834458 insertion comparing the sequenced products with the GenBank database.

Long-chain n-3 and n-6 polyunsaturated fatty acids are formed from the EFA linoleic acid and alpha-linolenic acid by sequential desaturation and elongation (Figure 1). There is a decreased promoter activity in FADS2 rs3834458 for this function [2]. As expected, the fatty acid profile of the plasma from subjects homozygous for the FADS2 rs 3834458 deletion [−/−] differed substantially from that of the other genotypes in that proportions of plasma total eicosapentaenoic acid (20:5 n-3), docosapentaenoic acid (22:5 n-3) and arachidonic acid (20:4 n-6) were significantly decreased. These findings are in line with earlier observations [3,8,9]. However, only about 8% of the dietary linoleic acid / α-linolenic acid go through the elongase/desaturase biosynthetic pathway to eicosapentaenoic acid and conversion to docosahexaenoic acid is extremely low (<0.1%) [10]. This may put into perspective the clinical relevance of the genotype findings. Moreover, the type of fat in the diet is reflected in serum fatty acids [11] and we had no knowledge on the habitual diet of the participants, which could have influenced the outcome. In spite of this limitation, it seems unlikely that the subjects homozygous for the FADS2 rs 3834458 deletion [−/−] would have had a diet that differed substantially from that of the other genotypes.

Earlier studies have shown that there are several intrinsic properties of lipoproteins that can affect their susceptibility to oxidation. Especially in supplementation studies, lipoprotein antioxidant content and fatty acid composition [12-15] have been shown to have an impact on oxidation parameters. We analyzed the fatty acid compositions of LDL, HDL_2_ and HDL_2_ particles and united the information in the fatty acid profiles into a single term – the peroxidizability index – which describes the combined reactivity of fatty acids towards reactive oxygen species [16]. The results of our experiments suggest that proportions of polyunsaturated fatty acids affected by FADS2 are related to the peroxidation index, oxidation rate and the amount of dienes formed during in vitro oxidation. This may also have implications in vivo since – although PUFA are considered to be beneficial in many aspects – they also have inflammatory and pro-oxidant properties.

## Conclusions

In summary, we report that compared to subjects with [T/T] or [T/-] in FADS2 rs 3834458, the formation of arachidonic acid and EPA were significantly decreased, as reported previously. What is new is that the theoretical peroxidizability index was also decreased in lipoproteins from these individuals. Consequently, when the isolated LDL and HDL were subjected to Cu^2+^-induced oxidation, the subjects with [−/−] showed decreased rate of appearance and the final concentration of conjugated dienes, compared to [T/T] and [T/-]. Thus, FADS2 genotype may play a role in peroxidation of lipoproteins.

## Methods

### Subjects

61 healthy subjects from the personnel and medical students of the Department of Medical Sciences of Tampere University and Tampere University Hospital volunteered. The age range of the subjects was 20 to 58 years. 33 were women and 28 were men. All participants filled in a questionnaire, where emphasis was given to their health status (diseases and use of medication) in addition to health related behaviour (smoking, use of alcohol and vitamins). The results of three of the participants were later removed from analysis because of reported bowel diseases. Thus, 58 subjects remained; 32 women and 26 men. All participants gave their written consent to the study. The study protocol was approved by the ethics committee of the Tampere University Hospital.

### Blood Samples

Fasting (12 h) blood samples were taken into suitable tubes (Vacuette, Greiner) from the antecubital vein in a sitting position after a 15-min rest using minimal stasis. Samples for the analysis of lipids, extraction of DNA and isolation of lipoproteins were taken into pre-chilled EDTA tubes, which were immediately placed in ice. Plasma was separated after centrifugation (Heraeus, 2000xg, +4°C). EDTA plasmas for isolation of lipoproteins were supplemented with sucrose (0.6% w/v final concentration). This procedure has been shown to preserve LDL from oxidation for at least two months and the oxidation curve does not differ from that of a fresh sample [17]. Lipids were analysed as described [6]. All samples were kept frozen at −70°C until analyzed.

### DNA isolation, FADS2 genotyping and Sequencing

Genomic DNA was extracted from peripheral blood leukocytes using a commercial kit according to the manufacturer’s instructions (Qiagen, Hilden, Germany). Insertion/deletion of T in FADS2 was genotyped with the use of 2-allele specific primers designed in such a way that the only difference between them was the insertion site at the very last nucleotide of their 3´ends. Prior to final genotyping the amplified products were confirmed by sequencing 8 clones from an individual who proved to be homozygous for insertion and 16 randomly selected clones from another one who was homozygous for deletion variant. After amplification, PCR products were run in 2% agarose gel electrophoresis. Two parallel PCRs were performed for each DNA sample. These contained primers: 5´CTAGGTGACGCCCTTCCTT 3´ (right), and either 5´GAGGTTCCGCAATTCTTTTCT 3´ (left) or 5´GAGGTTCCGCAATTCTTTTC 3´ (left). Amplification conditions were; 94°C 15 min, followed by 32 cycles of 94°C, 63°C and 72°C for 30 s each and final extension at 72°C for 5 min. For control sequencing, PCR was performed using primers D6Dsense (5’-GCC AGT TCC TCA TCG CCC CC-3’) and D6Dantisense (5’-TCC CTT CCC CAT GCT GCC TG-3’) [2]. PCR conditions consisted of 15 min at 94°C, followed by 33 cycles at 94°C 1 min, 69°C 1 min, 72°C 2 min, and final extension 72°C for 30 min. The primers produced fragments of 1059 bp. Prior to sequencing, amplified PCR products were cloned by using the TOPO TA (Invitrogen) cloning system. The automated ABI PRISM 3130 Genetic Analyzer (Applied Biosystems) and BigDye Terminator Cycle sequencing chemistry (Applied Biosystems) using M13(−20)F and M13R primers for determining DNA sequences on both strands. The partial 16S rDNA sequences of approximately 600 bp were edited and aligned using the Chromas 2.31 (Technelysium) and ClustalW sequence analysis software, and compared with those in the GenBank database by using the BLAST search tool (National Center for Biotechnology Information; http://www.ncbi.nlm.nih.gov/BLAST/).

### Isolation of lipoproteins and plasma fatty acid composition

Lipoproteins were fractionated by isopycnic density gradient ultracentrifugation using a Beckman SW40 Ti rotor in a Beckman L60 centrifuge (36000 rpm, 40 hours, 10°C) as previously described [6]. The fatty acid compositions were analyzed by capillary gas–liquid chromatography [6,7]. From fatty acid compositions, the peroxidizability index was calculated: (PI) = [(Σ mol% monoenoic FAs × 0.025) + (Σ mol% dienoic FAs × 1) + (Σ mol% trienoic FAs × 2) + (Σ mol% tetraenoic FAs × 4) + (Σ mol% pentaenoic FAs × 6) + (Σ mol% hexaenoic FAs × 8)] [14].

### Oxidation of lipoproteins

The susceptibility of mixtures of LDL and HDL_2_ or HDL_3_ subtractions to in vitro copper-catalyzed oxidation was assessed by continuously monitoring the production of conjugated dienes at 234 nm, as previously described [6,18]. Oxidation was started by adding 10 μl of CuSO4 to a final concentration of 1.65 μM Cu^2+^. The spectrophotometer was computer-operated (UVWinlab 2.1). This program also collected the absorbance data at 2-min intervals during the oxidation. Several characteristic oxidation indices were obtained from the resulting absorbance versus time curves [17,19].

### Data analysis

Results are expressed as mean ± SD unless otherwise stated. Statistical comparisons were made by Univariate General Linear Model, *T*-test and Mann–Whitney test using IBM SPSS software, version 20. Due to skewed distribution, plasma values of eicosapentaenoic acid and kinetic parameter of LDL + HDL_2_ oxidation rate were used as their logarithms but reported as their original results. A p value <0.05 was taken to be statistically significant.

## Competing interests

The authors report no competing interests.

## Authors’ contributions

TS, OJ and STN had substantial contributions to conception and design and interpretation of data and writing the manuscript. TK and TL had substantial contributions to conception and design. TS, JR and TK carried out the biochemical analyses. All authors read and approved the final manuscript.
